# Encapsulated Cells Expressing a Chemotherapeutic Activating Enzyme Allow the Targeting of Subtoxic Chemotherapy and Are Safe and Efficacious: Data from Two Clinical Trials in Pancreatic Cancer

**DOI:** 10.3390/pharmaceutics6030447

**Published:** 2014-08-11

**Authors:** J. Matthias Löhr, Stephan L. Haas, Jens C. Kröger, Helmut M. Friess, Raimund Höft, Peter E. Goretzki, Christian Peschel, Markus Schweigert, Brian Salmons, Walter H. Gunzburg

**Affiliations:** 1 Gastrocentrum, Karolinska University Hospital, Hälsovägen 1, SE-141 86 Stockholm, Sweden; E-Mails: matthias.lohr@ki.se (J.M.L.); St.Haas@gmx.de (S.L.H.); 2 Department of Medicine II, University Hospital Mannheim, University of Heidelberg, Theodor-Kutzer-Ufer, D-68135 Mannheim, Germany; 3 Institute of Diagnostic und Interventional Radiology, University Medicine Rostock, Ernst-Heydemann-Strasse 6, D-18057 Rostock, Germany; E-Mail: jens-christian.kroeger@med.uni-rostock.de; 4 Department of Surgery, Klinikum rechts der Isar der Technischen Universität München, Ismaninger Strasse 22, D-81675 Munich, Germany; E-Mail: helmut@friess.cc; 5 Abteilung für Gastroenterologie, Klinik und Poliklinik für Innere Medizin, Universität Rostock, Ernst-Heydemann-Strasse 6, D-18057 Rostock, Germany; E-Mail: Raimund.Hoeft@med.uni-rostock.de; 6 Chirurgische Klinik und Poliklinik, Medizinische Einrichtungen der Heinrich Heine Universität, Moorenstrasse 5, D-40225 Düsseldorf, Germany; E-Mail: pgoretzki@lukasneuss.de; 7 Medizinische Klinik und Poliklinik, Klinikum Rechts der Isar der Technischen Universität München III, Ismaninger Strasse 22, D-81675 Munich, Germany; E-Mail: christian.peschel@lrz.tu-muenchen.de; 8 Medizinische Klinik (Onkologie/Hämatologie) Campus Mitte, Universitätsklinikum Charité II, Schumannstrasse 21/22, D-10098 Berlin, Germany; E-Mail: info@onkologie-hellersdorf.de; 9 Austrianova Singapore Pte Ltd, Centros, Biopolis, Singapore; E-Mail: gunzburg@sgaustria.com; 10 Institute of Virology, Department of Pathobiology, University of Veterinary Medicine, Veterinaerplatz 1, A-1210 Vienna, Austria

**Keywords:** pancreatic cancer, cytochrome P450, cell therapy, encapsulation, ifosfamide, safety, efficacy, bioencapsulation, targeting chemotherapy, low dose, quality of life

## Abstract

Despite progress in the treatment of pancreatic cancer, there is still a need for improved therapies. In this manuscript, we report clinical experience with a new therapy for the treatment of pancreatic cancer involving the implantation of encapsulated cells over-expressing a cytochrome P450 enzyme followed by subsequent low-dose ifosfamide administrations as a means to target activated ifosfamide to the tumor. The safety and efficacy of the angiographic instillation of encapsulated allogeneic cells overexpressing cytochrome P450 in combination with low-dose systemic ifosfamide administration has now been evaluated in 27 patients in total. These patients were successfully treated in four centers by three different interventional radiologists, arguing strongly that the treatment can be successfully used in different centers. The safety of the intra-arterial delivery of the capsules and the lack of evidence that the patients developed an inflammatory or immune response to the encapsulated cells or encapsulation material was shown in all 27 patients. The ifosfamide dose of 1 g/m^2^/day used in the first trial was well tolerated by all patients. In contrast, the ifosfamide dose of 2 g/m^2^/day used in the second trial was poorly tolerated in most patients. Since the median survival in the first trial was 40 weeks and only 33 weeks in the second trial, this strongly suggests that there is no survival benefit to increasing the dose of ifosfamide, and indeed, a lower dose is beneficial for quality of life and the lack of side effects. This is supported by the one-year survival rate in the first trial being 38%, whilst that in the second trial was only 23%. However, taking the data from both trials together, a total of nine of the 27 patients were alive after one year, and two of these nine patients were alive for two years or more.

## 1. Introduction

Even though substantial progress has been made in unraveling the biology behind the development of pancreatic cancer, there has been little change in the success of treating this devastating tumor [1]. Pancreatic cancer is the fourth leading cause of cancer death in the USA with a median survival of only six months and a dismal five-year survival rate of 3%–5% [2,3,4]. Survival is better for those with malignant disease that is localized to the pancreas and is thus amenable to surgical resection, since at present, this offers the only chance of a cure. However, 80%–85% of patients present with advanced non-resectable tumors that respond only poorly to most chemotherapeutic agents [5]. Recent evidence suggests that pancreatic cancer develops at a similar speed to other tumor types, and it has been estimated that it takes about 12 years from the start of tumorigenesis to the formation of a primary pancreatic cancer, with a further seven years being needed for the seeding and development of metastatic disease [6]. Moreover, the primary tumor appears to be mix of genetically distinct subclones of which only one type gives rise to metastases, suggesting that the disease has a stem-cell origin [6,7].

The introduction of gemcitabine in 1997, which rapidly became the gold standard for the treatment of pancreatic cancer, was a milestone for this tumor type, as well as later on for other tumors, despite its modest effect on the median survival of patients suffering from this disease [3]. Since then, a number of combinations with gemcitabine, as well as new chemotherapeutic agents and/or gene and cell therapies are being developed as potential therapies to treat this tumor type (reviewed in [1,8,9,10]).

Ifosfamide is a chemotherapeutic agent with a long established history of clinical use that was approved some years ago for use in pancreatic cancer patients (Baxter Product Monograph: IFEX Ifosfamide for injection, [11]), but serious toxicity-related side effects have precluded its use at conventional doses (2.5–3 g/m^2^/day) [12,13]. Mid-range doses of ifosfamide (1.8–2 g/m^2^/day) result in less toxicity and partial treatment responses in the range of 60% with rare complete remissions [14,15,16], whereas low dose ifosfamide treatment (≤1.6 g/m^2^/day) results in non-severe toxicity, but is accompanied by, at best, rare, mostly partial treatment responses (≤33%) [17,18].

Like many chemotherapeutic agents, ifosfamide is a prodrug, *i.e.*, it is not tumor toxic *per se*, but upon metabolization by cytochrome P450 enzymes (mainly those that are expressed in the liver), it is converted into short-lived tumor toxic metabolites [19,20,21]. The short half-life of these metabolites in plasma, however, necessitates relatively high systemic levels of ifosfamide to achieve therapeutic, tumor toxic metabolite levels in the tumor. At the same time, these doses cause debilitating and unacceptable side effects [22]. Moreover, there are a number of different cytochrome P450’s, some of which produce metabolites that are neurotoxic due to the chloroacetaldehyde (CAA) catabolite [23,24,25]. Cytochrome 2B1 (CYP2B1), the rat isoform of the human cytochrome 2B6 [26], is particularly efficient at producing mainly only the tumor toxic and not the neurotoxic metabolites [27].

Some years ago, we and others argued that local tumor activation of ifosfamide, *i.e.*, in the proximity of the tumor, combined with relatively low-dose (1 g/m^2^/day) ifosfamide, should result in local high levels of cytotoxic activity and, at the same time, only minimal systemic side effects [28,29,30]. Local activation of prodrugs has been achieved in animal models of pancreatic cancer after delivery of enzyme encoding genes (suicide genes) using virus vectors [9]. We have taken a different approach in that we have used genetically modified allogeneic cells to over-express CYP2B1 at the site of the tumor. This has necessitated the encapsulation of these cells, so that they are confined to the site at which they should act, as well as to protect them from the host immune system and to also prevent their replication, so that they are not killed by the activated prodrug [28,31,32].

**Figure 1:**
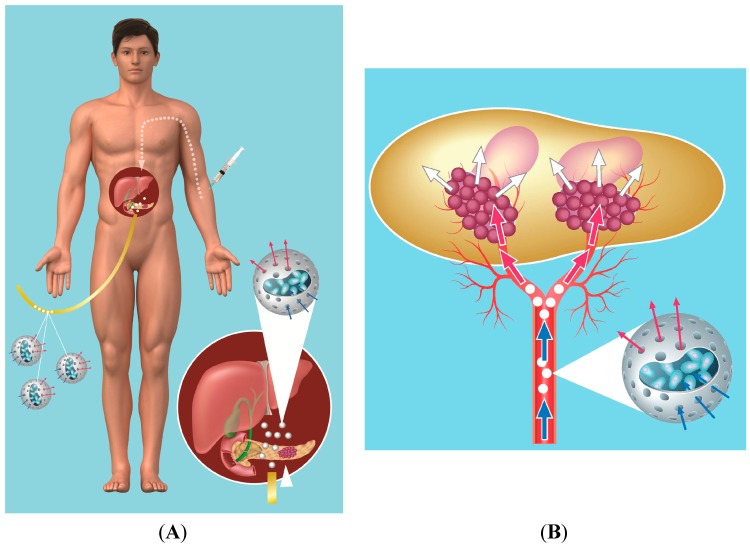
(**A**) schematic of placement of capsules in vessels leading to the pancreatic tumor using supraselective angiography with a catheter being inserted into a vessel in the groin, followed by low-dose ifosfamide administration given intravenously (IV); (**B**) schematic of ifosfamide (prodrug) conversion by encapsulated cells. Ifosfamide (blue arrow) delivered systemically enters the porous capsules and penetrates the encapsulated cells, where it is converted by the cytochrome P450 enzyme to its active form (red arrow). The short-lived activated ifosfamide (white arrow) exits the cells and leaves the capsules to bathe the tumor.

The cells have been encapsulated in cellulose sulfate capsules [33], since this biomaterial is inert. Cells within the capsule survive and thrive for long periods, and upon implantation, they show good biocompatibility. Moreover, due to their flexibility and robustness, the cell-containing capsules (Figure 1A) can easily be injected through a needle or catheter without bursting, and they can specifically be delivered to the pancreas by the tumor vasculature under angiography (Figure 1B) [34,35,36].

Initial data from a first clinical trial, approved and performed in Germany [36], has been published previously [37,38,39]. This article aims to provide an update and overview of the safety and efficacy results obtained in patients from two clinical trials, the first involving 14 patients treated at a single clinical center and a second one involving 13 patients treated at four clinical centers in two European countries.

## 2. Results of Clinical Trials

All but two patients in the first trial were diagnosed with Stage IV disease, with the other two having Stage III disease. In the second study, 12/13 of the patients were classified as Stage IV with one patient being diagnosed as Stage III.

The first trial involved 14 patients were treated at one clinical center, the Department of Internal Medicine, Division of Gastroenterology and Endocrinology, University of Rostock, and all of them received 1 g/m^2^ ifosfamide. The patients in the second trial were treated at four different centers and received 2 g/m^2^. Thus, taken together, a total of 30 patients (17 males and 13 females) were enrolled in the two studies. The age ranged from 43 to 78 years old with an average age of 61.5 years of age. However, in the first study, three patients could not be treated (two due to infections and one because angiography was not successful), and so, a total of 27 patients (16 male and 11 female) have actually been treated to date with the encapsulated cells followed by ifosfamide.

### 2.1. Objectives of the Two Clinical Trials

The primary aim of the first phase 1/2 trial was to assess the safety and tolerability of the angiographic instillation of encapsulated, genetically modified cells into the tumor in patients with non-resectable pancreatic carcinoma Stages II to IV. The secondary objective was to obtain a description of the clinical effects of the cell-therapy compared to patients given the best available treatment in the years prior to the study (historical data) with respect to response, survival, failure-free survival and progressive disease. This trial protocol was approved (20 March 1998, Protocol No. 23) by the working party for gastrointestinal oncology (AGO) of the German Gastroenterology Society (DGVS), as well as by the state ethics committee (approval on 7 May 1998, Reg. No. I MPG 1/98; registration on 10 July 1998, AMUSt 20a/610.1) and the federal ethics committee (approval on 6 July 1998, Az.: 854.052, Protocol No. 16; registration on 6 July 1998, Reg. No. 4013824). It was registered with the state authority, as well as the federal authority.

The primary objective of the second, phase 2 clinical trial was to determine tumor response rate defined by stable disease (SD), partial remission (PR) and complete remission (CR) and the clinical benefit (Karnofsky score, body weight, pain) of the treatment with encapsulated cells followed by ifosfamide in patients with non-resectable pancreatic cancer. The secondary aim of this study was to determine the time to progression, tumor response, duration of partial or complete remission, time of symptom-free survival, survival time and quality of life. In addition, another secondary aim of this study was to evaluate the safety and tolerability of the treatment regimen with special attention being paid to the appearance of pancreatitis or immediate-type allergic reactions. The protocol was approved by the state ethics committee (Votum der Ethikkommission der Ärztekammer Mecklenburg-Vorpommern, 8 November 1999), as well as by the German Medical Association committee (9 July 1999, registration number 4015446) for somatic gene therapy (Votum des Wissenschaftlicher Beirats der Bundesärztekammer/Kommission Somatische Gentherapie) and was registered with the Mecklenberg-Vorpommen Health office (Gesundheitsamt) and the Federal Institute for Drugs and Medical Devices (Bundesamt für Arzneimittel und Medizinprodukte (BfArM)). It was also approved by the working party for gastrointestinal oncology (AGO) of the German Gastroenterology Society (DGVS).

In the first study, an appropriate artery leading into the tumor (Table 1) could be supra-selectively cannulated in 14 of the 17 patients entering the study. Two patients developed severe infections before the start of the trial and had to be treated by other means, whilst angiography was not successful in one patient due to an unusual blood vessel architecture. In contrast, in the second study, all patients could be given capsules via supra-selective angiography (Table 1). Thus, when considering both trials together, a final total of 16 males and 11 females were successfully administered encapsulated cells. However, whereas in the first study, all patients, except one (*i.e.*, 13/14 patients), received the planned dose of 300 cell-containing capsules (with one patient only receiving 250 capsules); the number of capsules given in the second trial varied from 160 up to 450 capsules (Table 2).

All of the patients in the first trial received ifosfamide at a dose of 1 mg/m^2^, whereas a dose of 2 mg/m^2^ was given in the second trial according to the label instructions, *i.e.*, on Days 2, 3 and 4 and Days 23, 24 and 25 post capsule administration, plus an isodose of mesna to protect against urotoxicity. Table 2 shows an overview of the patients, as well as the location of the centers involved in the clinical trials, the dose of encapsulated cells that the patients received and the subsequent dose of ifosfamide. The patients were then followed for a variety of parameters associated with safety, as well as efficacy.

**Table 1:** Vessels targeted for encapsulated cell instillation.

**Phase I/II Trial**	**Number of Patients**
A. pancreatica dorsalis	−2 patients
A. pancreaticoduodenalis	−4 patients
Rami anterior of A. pancreaticoduodenalis + accessory branch + branch of A. pancreatico-dorsalis	−1 patient
Anterior pancreatic arcade	−2 patients
Posterior pancreatic arcade	−1 patient
A. pancreatico-duodenalis superior	−1 patient
Dorsal arcade of pancreatic head	−1 patient
A. gastroduodenalis inferior	−1 patient
Rami pancreatici of A. lienalis	−1 patient
**Phase II Trial**	**Number of Patients**
Anterior and posterior pancreatic arcade + tumor vessel which infiltrated the liver	−1 patient
A. pancreaticoduodenalis inferior and transversa accessoria	−1 patient
A. pancreaticoduodenalis inferior and A. pancreatica dorsalis	−1 patient
A. pancreatico duodenalis inferior	−1 patient
A. pancreaticoduodenalis superior	−1 patient
A. pancreaticoduodenalis superior ramus posterior + ramus ventralis, A gastroduodenalis	−1 patient
A. pancreaticoduodenalis caudal branches I and II + transversal branch	−1 patient
A. pancreatica dorsalis	−1 patient
A. mesenterica superior	−1 patient
A. gastroduodenalis	−2 patients
A. pancreatica transversalis	−2 patients

A. is short for arteria.

**Table 2:** Patient overview from the two clinical trials.

Center	Patients				
	Screened	Enrolled	Treated	No. Capsules ^+^	Ifosfamide Dose
Rostock ^1^	51	17	14	300 *	1 g/m^2^
Rostock ^2^	8	7	7	221	2 g/m^2^
Berlin ^2^	5	1	1	250	2 g/m^2^
Munich ^2^	6	3	3	343	2 g/m^2^
Berne ^2^	2	2	2	300	2 g/m^2^
Total	72	30	27	Mean = 244 ^$^	

^1^ Phase1/2 center; ^2^ phase 2 center; ^+^ each capsule contained 1 × 10^4^ cells; * one patient received 250 capsules; ^$^ if the phase 2 trial is considered alone, then on average, each patient received 264 ± 70 cell-filled capsules (median: 250).

### 2.2. Safety Parameters and Results

The safety analysis concerned three aspects of the treatment, *i.e.*, angiography, capsule instillation and chemotherapy.

On average, angiography took approximately 40 min [37]. Most frequently, the encapsulated cells were instilled through the Arteria pancreaticoduodenalis (A. gastroduodenalis) (Table 1). More than one vessel had to be used for instillation of the encapsulated cells in only one patient in the first trial, but this was necessary in five patients in the second trial. None of the 14 patients had any acute complaints during angiography. The quality of the angiographic intervention was estimated as “good” in 13 patients (92.9%) and as “intermediate” in one patient (No. 12). The administration of the encapsulated cells was also well tolerated in the second trial.

In the first trial, acute toxicity or allergic reactions were not observed after instillation of the encapsulated cells and in the follow up neither abdominal symptoms nor were blood biochemical changes suggestive of pancreatitis observed. There were also no signs of allergic reactions (eosinophil count) or hemorrhagic cystitis (urine sticks). One patient showed increased serum lipase levels 15 days after encapsulated cell instillation, but this was attributed to the disease rather than to capsule instillation.

In the second trial, there were also no signs of allergic reactions (eosinophil count) or hemorrhagic cystitis (urine sticks) after the encapsulated cells had been implanted. Two patients had increased lipase at baseline; in one patient, lipase measurements decreased to a level that was no longer considered as clinically relevant. In another patient, whose lipase measurements fluctuated between normal and elevated levels, the final measure was assessed as clinically not relevant. However, a CT scan of this patient suggested a possible pancreatitis in three assessments after therapy (Weeks 7, 10 and 14). Nevertheless, no clinical report for pancreatitis is available, nor was a specific treatment initiated.

In conclusion, the safety analyses did not reveal any clinically relevant risk associated with the instillation of encapsulated cells. Especially, there were no clinically relevant signs of pancreatitis, and no allergic reactions were registered.

However, with respect to the third component of the treatment, the dose of 2 g/m^2^ of ifosfamide was found, as expected, to be toxic in the majority of patients in the second phase 2 trial, necessitating one patient to receive a reduced dose during the second cycle. In contrast, the dose of 1 g/m^2^ was well tolerated by all patients in the first trial.

The toxicity profile experienced by patients in the phase 2 trial reported in this paper is in line with the findings of Ajani and colleagues who performed a phase 2 trial in 31 patients with pancreatic cancer who had not received prior chemotherapy and who were treated with a median ifosfamide dose of 2 g/m^2^/day (range, 1.5 to 2 g/m^2^/day) administered intravenously (IV) over one hour for five consecutive days with mesna [16]. The most common toxic effects included nausea and vomiting, malaise, anorexia and mild hematuria. Mesna offers an adequate protection against uroendothelial injury caused by ifosfamide. However, unlike the study reported here, the data from Ajani and colleagues suggest that ifosfamide alone is only marginally active against cancer of the pancreas and appears to be of minimal value in the treatment of patients with this tumor. Indeed in the Ajani study, only one patient of the 30 evaluable patients achieved a complete remission (more than 26 months), and another patient had a partial remission (four months). The median duration of survival of all patients from the start of ifosfamide therapy was only three months (range, one to more than 26 months) [16]. A more recent study that employed ifosfamide 2.5 g/m^2^ and mesna together with mitomycin C was prematurely stopped because of a median survival of only 3.7 months that was accompanied by high levels of Grade 3–4 toxicity [40]. Taken together, these studies and our two studies suggest that the encapsulated cells improve median survival, but that benefit is achieved by using 1 gm/m^2^/day ifosfamide without the toxicities experienced by patients receiving 2 g/m^2^/day.

### 2.3. Serious Adverse Events

Although 11 serious adverse events (SAEs) were recorded in seven patients during the study period for the phase 1/2 clinical trial, none of these SAEs were deemed to be treatment related (*i.e.*, due to instillation of the encapsulated cells or to the ifosfamide treatment) and were rather attributed to the underlying disease and/or the effects thereof [37,38]. Importantly, the delivery of the encapsulated cells to the vasculature leading to the tumor did not result in any obvious allergic or inflammatory responses, and none of the patients developed pancreatitis at any time during the course of the clinical study. Elevated amylase levels were detected in some patients, but they appeared to be a result of the tumor infiltration of the pancreas and limited obstructive (chronic) pancreatitis, and no further increase was measured after the angiographic procedure to place the encapsulated cells [38]. A single AE in one patient (increased lipase activity observed on Day 15 after capsule instillation) may have been possibly related to the angiographic administration procedure [38].

A total of 16 SAEs in eight patients was documented in the second trial, including the three SAEs leading to death (Table 3). A detailed description of each SAE from this trial is given in Table 3, the most common of which were obstructions. Importantly, none of these SAEs were attributed to the instillation of the encapsulated cells. Patient 2-5 showed signs of neurological impairment, *i.e.*, drowsiness, nocturnal enuresis, mild somnolence, on the second day of the first chemotherapy cycle. There were no other signs of neurological dysfunction. He was treated with additional hydration (infusions with isotonic saline and glucose). This SAE was deemed to be due to the treatment with ifosfamide. From the SAE analysis, there was no evidence of pancreatitis or any allergic responses at any time during the course of the study.

All of the patients in the phase 2 trial experienced between five and 19 adverse events (AEs), with a median number of nine AEs per patient. There were 6 AEs (5.6%) rated as life-threatening, 10.2% as severe, 28.7% as moderate and 53.7% as mild. Importantly, none of the AEs was thought to be related to the administration of the encapsulated cells, but 44 (mainly mild to moderate in intensity, only two severe) were related to ifosfamide at the higher 2 g/m^2^ dose. The most frequent AEs were the toxicities of the chemotherapy, namely alopecia (in 76.9% of all patients), anemia (69.2%), leucopenia (61.5%), vomiting and nausea (53.9% each) or encephalopathy (23.1%). Other AEs were new or worsened symptoms of the underlying disease, like abdominal pain (53.9%), weight decrease (30.8%), bile duct stricture or intestinal obstruction (23.1%). Compared to the baseline status, a total of 65 events fulfilled any of the National Cancer Institute (NCI) common toxicity criteria (NCI toxicities); of these, 46.2% had Grade 1, 40% Grade 2, 9.2% (six events) Grade 3 and 4.6% (three events) Grade 4 (multiple counts per patient possible). Decreased leucocyte counts and alopecia (in 76.9% of all patients), nausea (61.5%), vomiting (53.9%), decreased granulocyte counts (46.2%) and decreased hemoglobin (38.5%) were documented. Grade 3 nausea was experienced by two of the patients during chemotherapy; Grade 3 leucopenia was noted in four patients. Grade 4 toxicities were decreased leucocytes and increased serum blood urea nitrogen (BUN) in one patient and decreased granulocytes in another. In total, five patients experienced Grade 3 or Grade 4 NCI toxicities.

**Table 3:** Documented deaths and serious adverse events (SAEs) in the phase 2 trial.

Patient No.	Days After Instillation	Description
*Death ^#^*		
2-1	96	Liver metastases, death for unknown reason
2-2	66	Occult bleeding from eroded tumor vessel
2-5	36	Tumor progression
*Other SAEs*		
2-1	11	Complete obstruction of duodenal passage by tumor
	22	Bile duct obstruction by tumor
2-2	48	Incomplete obstruction of duodenal passage by tumor
2-3	71	Elective hospitalization to change bile duct stent
	113	Gastric outlet stenosis
2-5	3	Somnolence in the context of an ifosfamide-induced encephalopathy
2-6	5	Stent occlusion
	14	Duodenal stenosis
	36	Acute renal failure
2-10	52	Incomplete obstruction of the bile duct by the tumor, with fever, leukocytosis, cholestasis
2-12	111	Peritoneal carcinomatosis in the context of a planned relaparotomy after marked improvement of the pancreatic tumor
2-13	90	Jaundice
	105	Liver abscess

^#^ Formally, deaths are also SAEs.

### 2.4. Tumor Reductions

The size of the primary tumor was measured prior to starting the treatment and at Weeks 10 and 20 post-treatment [37,38]. The tumor did not grow any further during this observation period in any of the treated patients in the phase 1/2 trial (Table 4). Two of the 14 patients treated in this trial (Patients 2 and 8) showed a partial response (PR), characterized by a more than 50% reduction in tumor volume (Table 4). The remaining 12 patients showed a stable disease (SD) with tumor sizes in the range of 50%–125% of the initial size (Table 4) [37]. Of these 12 patients, two demonstrated a minor response (MR), *i.e.*, tumor reduction by 25% to 50%.

In the phase 2 trial, no partial remissions were observed (Table 4), but four patients did show tumor size reductions, whilst the other four patients showed tumor growth, and the remaining five patients were classified as “stable disease” during the period over which they were followed up after chemotherapy. The most pronounced decrease of tumor size was 47%, which was observed in Patient 2-4, but this improvement was not stable and less pronounced (only 23%) at a later assessment in Week 16.

Thus, of the 27 patients that were evaluable from both of our trials, the majority (16) showed stable disease. There were two patients in the first trial that showed partial responses where their tumors were reduced in size by more than 50%, and four additional patients from the second trial showed a minor response (reduction in size between 25% and 50%), with one of these patients (2-4) showing a 47% reduction in tumor size at Week 10, although this reduction was not maintained at Week 16. The tumor status for a further four patients in the second trial showed progressive disease, and the status of the remaining five patients could not be determined, either due to death or the patient dropping out of the study.

**Table 4:** Patient disease stage and response overview from both trials.

Patient	TNM	Stage	Metastases	Tumor	Survival Weeks ^$^	Notes
1	T4N1Mx	IV	n	SD	102	
2	T4N1Mx	IV	n	PR	39	
3	TN4xMx	IV	n	SD *	64	
4	T3NxM1	IV	y	SD	29	
5	T3N1M1	IV	y	SD *	67	
6	T4N1M1	IV	y	SD	20	
7	T4N1M0	IV	n	SD	65	
8	T4N1M1	IV	y	PR	28	
9	T3NxMx	IV	n	SD	44	
10	T3N0M0	III	n	SD	33	
11	T4N1M0	IV	n	SD	112	
12	T4N1M1	IV	y	SD	6	
13	T3N0M0	III	y	SD	35	
14	T4N1Mx	IV	n	SD	41	
2-1	T4N1a/bM0	IV	y	PD	14	
2-2	T4N1a/bM0	IV	y		9	
2-3	T4N1a/bM0	IV	y	PD	34	
2-4	T4N1bM0	IV	y	SD *	47 ^#^	Single infusion ^^^
2-5	T4N1bM0	IV	y		5	No 2nd ifosfamide cycle
2-6	T4N1bM0	IV	y	SD *	67 ^#^	Three cycles ^^^
2-7	T4N1a/bM0	IV	n	SD *	114 ^+^	Two cycles ^^^
2-8	T4N1bM0	IV	n		57 ^#^	Two cycles ^^^
2-9	T4N0M0	IV	y	PD	26 ^#^	
2-10	T3N1M0	III	y		27 ^#^	
2-11	T4N0M0	IV	y	SD	20 ^@^	
2-12	T4NXM0	IV	y	SD	56 ^#^^,@^	
2-13	T4NXM0	IV	n	PD	26	

T, describes the size of the original (primary) tumor and whether it has invaded nearby tissue; N, describes nearby (regional) lymph nodes that are involved; M, describes distant metastasis (spread of cancer from one part of the body to another); ^$^ Survival weeks; ^#^ measured from capsule instillation; n, no; y, yes; SD *, minor responses, *i.e.*, between 25% and 50% reduction in tumor volume; PR, partial response; PD, progressive disease; ^^^ gemcitabine given as a follow on treatment; ^@^ peritoneal carcinomatosis diagnosed on Day 111; ^+^ this patient was still alive after 114 weeks.

### 2.5. Median Survival

**Figure 2:**
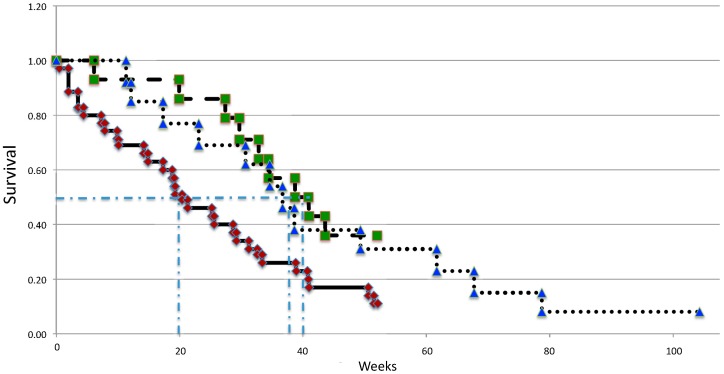
Kaplan–Meier curves describing the survival of patients from the phase 1/2 trial (green boxes), the phase 2 trial (blue triangles) and an age and disease stage matched historic control group receiving the best available standard care (red diamonds).

The data from the Kaplan–Meier survival curves for patients in each of the two trials are shown in Figure 2 and the median survival and one-year survival rates in Table 5. As can be seen, the median survival in the phase 1/2 trial was 40 weeks (range: 6–102 weeks), whereas in the second trial, this was 38 weeks (range: 5–114 weeks). Interestingly, the majority of the survival benefit was exhibited early on, with the patients eventually succumbing to the disease at a similar rate to the historic controls. This suggests that a prolongation of the survival benefit might be achieved if additional cycles of ifosfamide are given to the patients. The one-year survival rate in the first trial was 36%, which is twice that of gemcitabine (Table 5). In contrast, the one-year survival for the second trial was 23% and is possibly attributable to the side effects of the higher dose of ifosfamide. The small sample size of our studies, however, should be noted, and this limits the interpretation of the one-year survival data.

**Table 5:** Summary of survival data for patients receiving encapsulated cells followed by low-dose ifosfamide.

Treatment	*N*	Phase	Median Survival	1 Year Survival	Notes
Encapsulated cells					
+1 g/m^2^/day ifosfamide	14	1/2	10 months	36%	Single center
+2 g/m^2^/day ifosfamide	13	2	9.5 months	23%	Multiple centers
Control	36	n/a	5 months	11%	Historical data
Gemcitabine	63	3	7 months ^#^	18%	Pivotal study

n/a, not applicable; ^#^ in a meta-analysis, gemcitabine gave a median survival of 5.4–5.6 months [41].

### 2.6. Quality of Life

Both trials included an assessment of the quality of life of patients using the core questionnaire for cancer patients, QLQ-C30, and a German quality of life scale for pancreas patients [42], which were completed by the patient. This was done independently; thus, the assessment of the quality of life data did not interfere with the routine documentation of the adverse events that were reported by the patient. Quality of life data were available from the baseline evaluation for all 14 patients and for the analysis of change from eight patients in the first trial, and the analysis was performed strictly according to the European Organization for Research and Treatment of Cancer (EORTC) recommendations [43]. In the first trial, evidence was obtained that there was a general increase in quality of life, including no requirement for increased pain medication. In the second trial, data from the disease-specific module of the questionnaire revealed that pain during the night decreased, but patients felt themselves to be less attractive and lost interest in sex. All other scales and items remained fairly stable during the observation period; no further improvements in quality of life data were observed, besides reduced pain during the night.

## 3. Discussion

A variety of chemotherapeutic agents are used to treat cancers, and pancreatic cancer is no different. Gemcitabine has generally replaced 5-fluorouracil as the chemotherapeutic of choice, but in terms of survival, relatively little has been gained by this. Ifosfamide has been approved for pancreatic cancer, but is rarely used. A major limitation of many chemotherapies, including gemcitabine, 5-flurouracil and ifosfamide, is the associated toxicity and consequent severe side effects in which continued use results.

Recently, it has been shown that two chemotherapy drugs used for pancreatic cancer treatment (gemcitabine and 5-fluorouracil) are able to influence the immune response in a way that facilitates tumor growth, at least in animal models. Although 5-fluorouracil and gemcitabine activate the *NOD-like receptor family, pyrin domain containing 3* (*NLRP3*) inflammasome complex within myeloid-derived suppressive cells and selectively eliminated them in tumor-bearing rodents, they also promote the release of IL-1β and the development of pro-angiogenic IL-17-producing CD4 T-cells, thus preventing a robust immune response against the tumor [44].

We sought to use cell therapy to allow lower doses of chemotherapy to be used, while at the same time targeting the chemotherapy to the tumor. This is achieved by localizing cells that have been modified to activate the chemotherapeutic to blood vessels immediately upstream of the pancreatic cancer.

Here, we present the results from two trials that support the notion that the delivery of these encapsulated cells is safe and well tolerated without evidence of inflammatory disease, like pancreatitis. The capsules are made of a biologically inert natural polymer that is not only biocompatible and acts as a localization device, but also protects the enveloped cells from the patient’s immune system. After angiographic instillation of the encapsulated cells at the site of the tumor, low dose ifosfamide is administered systemically.

The two trials described here differed primarily in the dosage of ifosfamide that was given. In the first study, this was 1 g/m^2^ and was not associated with any toxicity beyond Grade II that was generally indistinguishable from disease symptoms. In the second study, the dose of ifosfamide was increased to 2 g/m^2^, and five of the thirteen patients in this study experienced Grade 3 or Grade 4 NCI toxicities.

The increased dosage of chemotherapy did also not bring any additional benefit with respect to parameters of efficacy, such as tumor reduction, improvement of median survival or quality of life. Thus, we can conclude that the lower dose of 1 g/m^2^ gave a better tolerability and associated therapeutic benefit than the higher dose.

An encouraging finding in the second trial is that the encapsulated cells could be delivered to the vasculature leading to the pancreatic cancer in four independent centers. Although skill is required to deliver the encapsulated cells via supra-selective catheterization of these vessels, this demonstrates the general applicability of this treatment. In the meantime, we have also developed a more robust, GMP-compliant manufacturing of encapsulated cells, as well as a freezing protocol that allows the encapsulated cell product to be shipped all over the world and also to be stored for at least one year at −80 °C [45,46], which should facilitate the use of this product.

The mechanism of action of this treatment has been shown to be due to the metabolization of ifosfamide (and other members of the oxalophosphamide family, such a cyclophosphamide) to the short-lived products, phosphoramide mustard and acrolein. Phosphoramide mustard is a DNA alkylating agent and is thought to be a tumor toxic agent. Although DNA in all cells that come into contact with phosphoramide mustard becomes alkylated, it is only when a cell with a threshold amount of damage is about to divide that it receives signals to die [28,29,30]. This is the basis for the tumor selectivity of this agent, but also is responsible for the side effects seen in organs and tissues where cell division is ongoing. It is also the presumed reason why encapsulated cells are unaffected by the DNA alkylation, since these cells are not proliferating and dividing [32]. Our previous studies have shown that, surprisingly, cells with alkylated DNA appear to die by necrosis rather than by apoptosis [47], although more recent studies have suggested that this can be more complicated, since in the absence of phagocytosis, apoptotic cells become necrotic [48]. This may be of relevance, since it has recently been reported that apoptotic cells are significantly more immunogenic than necrotic cells, even though both forms are identical in antigenic composition. This is not due to necrotic cells being immunosuppressive or tolerogenic, and debris from both apoptotic and necrotic cells are taken up by antigen-presenting cells in a similar manner. Moreover, priming of naive T-cell responses is equivalent for apoptotic and necrotic cells. However, the CD8^+^ T-cells activated by apoptotic cells amplify and augment effector functions, while those primed by necrotic cells do not. In contrast, apoptotic and necrotic cells elicit equivalent CD4^+^ T-cell priming, accumulation and function. Buckwalter and Srivastava reported that the deficiency in CD8^+^ T-cell function elicited by necrotic cells can be overcome to varying degrees by anti-CD40 antibody and ligands for TLR4 (Toll like receptor 4) and TLR9, suggesting potential add-on therapies if indeed necrosis is the mechanism by which tumor cells are dying [49].

A second possible contributing mechanism of action could be via stimulation of the immune system and anti-angiogenic effects, as has been described for metronomic (low-dose, long-term and frequently administered) chemotherapy. Another member of the oxalophosphamide family, cyclophosphamide, is the most widely-explored agent in such an approach [50]. Cyclophosphamide is related to ifosfamide, and it can also be activated by cytochrome P450 enzymes [30]. We have performed a clinical trial in dogs using encapsulated cells to locally activate this chemotherapeutic after peritumoral injection [8,51,52]. Tumor reductions were also observed in this clinical trial in companion animals [52], supporting the notion that the mechanism of action is not due to a simple obstruction of the blood vessels leading to the tumor, since in this study, the capsules were administered by injection directly into the tissues round the tumor site. Further, CT-scans taken during and after angiography in our human pancreatic cancer trials showed the patency of the vessels after instillation of the capsules.

The chemotherapeutic agent, ifosfamide, has been shown to have potentially therapeutic effects for pancreatic cancer [22]. In a phase 2 trial in which 1.6 g/m^2^/day ifosfamide was administered for five days to 21 evaluable patients, seven cases of stable disease with mostly non-severe, Grades 1–2 toxicity were reported [17]. In another study, up to 1.5 g/m^2^/day ifosfamide were given as a 10-day continuous IV infusion to patients with various tumor types, and one of six patients with pancreatic cancer showed a partial response, with a second showing a tumor reduction of 45% [18]. The major side effects observed were leukopenia with granulocytopenia, whilst subjective side-effects included nausea/vomiting and fatigue (probably related to neurotoxicity). More encouraging clinical effects have been observed in other trials, where medium doses of ifosfamide (1–2 g/m^2^) have been investigated, but this is accompanied by medium-grade toxicity profiles. In an initial study by Gad-El-Mawla and colleagues, where 2 g/m^2^ were given for five days, all but two patients developed hemorrhagic cystitis. However, there were six partial responses in 10 patients [14]. A further study revealed that of 25 patients receiving daily doses of 1.8 g for five days, one patient showed complete remission and 14 patients showed partial remission [15]. However, these patients suffered from Grade 3 alopecia (100%), Grade 1 anemia (100%) and leukopenia (30%). Thus, it is to be expected that higher doses (2–3 g/m^2^/day) of ifosfamide will be associated with possibly unacceptable levels of toxicity.

Most recently, trial data for two more promising treatment regimes for pancreatic cancer have been reported. The first of these was a phase 2–3 trial of a combination chemotherapy regime consisting of oxaliplatin, irinotecan, fluorouracil and leucovorin (also known as FOLFIRINOX). The median overall survival was 11.1 months for patients receiving FOLFIRINOX, which represented an improvement of 4.3 months over gemcitabine (Table 6). However, the safety profile was less favorable than that of gemcitabine, and treatment was associated with a higher incidence of Grade 3 or 4 toxicities [53]. In 2013, von Hoff and colleagues reported the results of a phase 3 study of a combination therapy consisting of protein-bound paclitaxel (commonly referred to as Abraxane) plus gemcitabine. In this study, the median survival was 8.5 months, as compared to 6.7 months for gemcitabine (Table 6), and again, treatment-related adverse events of Grade 3 or higher were reported for the combination therapy [54].

The median survival that was observed in the two clinical trials summarized in this publication is 10 months, and treatment-related side effects are limited, particularly in the first study employing 1 g/m^2^/day (Table 6). The data suggest that future trials, possibly using additional cycles of low (1 g/m^2^) ifosfamide, may be warranted, since the encapsulated cells maintain viability in animal studies, even after a number of cycles of ifosfamide. Moreover, the inclusion of other chemotherapeutic or prodrug activating enzymes or even other antitumor agents, such as anti-angiogenic agents, may allow patient-specific tumor treatments that circumvent any pre-existing or treatment-induced resistance [55].

Pancreatic cancers generally consist of heterogeneous cells and include cells with properties of stem cells that express aldehyde dehydrogenase (ALDH), an enzyme that is associated with resistance to chemotherapy [56,57]. Recent publications suggest that use of disulfiram, an irreversible inhibitor of ALDH [58], or dasatinib, a potent inhibitor of SRC family kinases and ABL kinases [59], in combination with chemotherapeutic drugs may be able to address this problem and slow the growth of pancreatic tumors. Such agents thus may be useful in combination with encapsulated cells plus low-dose ifosfamide.

**Table 6:** Summary of recent trial data for pancreatic cancer treatments.

Treatment	Control	Phase	Median Survival		One-Year Survival		Side Effects	FDA Approval
			Therapy	Control	Therapy	Control		
gemcitabine	5-fluorouracil	3	5.7	4.2	18%	2%	more favorable than 5-fluorouracil	1996
gemcitabine + erlotinib hydrochloride (Tarceva)	gemcitabine	3	6.4	6	24%	19%	less favorable than gemcitabine	2005
gemcitabine + protein-bound paclitaxel (Abraxane)	gemcitabine	3	8.5	6.7	35%	22%	less favorable than gemcitabine	2013
FOLFIRINOX *	gemcitabine	2/3	11.1	6.8	48.4	20.6	less favorable than gemcitabine	n/a
cell encapsulation + ifosfamide	5-fluorouracil	2	10	5	36%	18%	more favorable than 5-fluorouracil or gemcitabine #	n/a

* Oxaliplatin, irinotecan, fluorouracil and leucovorin; ^#^ five patients experienced Grade 3 or Grade 4 NCI toxicities with a higher dose of 2 g/m^2^ and a median survival of 9.5 months.

## 4. Conclusions

The results of two clinical studies are reported and compared in this paper. Both revealed the safety and feasibility of delivering encapsulated cells over-expressing a cytochrome P450 enzyme to blood vessels leading to pancreatic cancers, followed by low-dose ifosfamide treatment. The second trial involved four clinical centers and showed that the procedure and treatment can thus be generally performed, regardless of location or staff. The safety of both the delivery procedure and the long-term instillation of the encapsulated cells has been shown in 27 patients. The efficacy profile of this treatment was similar regardless of whether 1 g/m^2^/day or 2 g/m^2^/day doses of ifosfamide were given, but the ifosfamide-associated side effects were more severe when 2 g/m^2^/day was administered. Taken together, these data suggest that additional later stage clinical trials of this unique therapy are warranted.
